# isiXhosa Translation of the Patient Health Questionnaire (PHQ-9): A Pilot Study of Psychometric Properties [Stage 1]

**DOI:** 10.3389/fpsyt.2022.840912

**Published:** 2022-02-28

**Authors:** Arish Mudra Rakshasa-Loots, Barbara Laughton

**Affiliations:** ^1^Edinburgh Neuroscience, School of Biomedical Sciences, College of Medicine and Veterinary Medicine, The University of Edinburgh, Edinburgh, United Kingdom; ^2^Department of Paediatrics and Child Health, Family Centre for Research With Ubuntu (FAMCRU), Stellenbosch University, Cape Town, South Africa

**Keywords:** HIV-associated depression, major depressive disorder (MDD), psychometric testing, translation, multicultural tools

## Abstract

Depression is a debilitating illness, and stigma associated with it often prevents people from seeking support. Easy-to-administer and culturally-specific diagnostic tools can allow for early screening for depression in primary care clinics, especially in resource-limited settings. In this pilot study, we will produce the first open-access isiXhosa-language version of the nine-item Patient Health Questionnaire (PHQ-9), a well-validated measure of depression incidence and severity, using a transcultural translation framework. We will validate this isiXhosa PHQ-9 in a small sample of adolescents living with HIV in Cape Town, South Africa who speak isiXhosa at home. Participants have previously completed the ASEBA Youth Self Report (YSR) form, and responses from the YSR will be used as a gold standard to validate the isiXhosa PHQ-9. If validated through this Registered Report, this isiXhosa PHQ-9 may be an invaluable culturally-specific tool for clinicians serving Xhosa people in identifying clinical or sub-clinical depression.

## 1. Introduction

“If I show you where I'm struggling, I feel you have been exposed to my weakness,” said Siyanda, a young Xhosa man, in relation to the cultural expectation for Xhosa men to manage mental health issues without seeking support (1, p.56). The constellations of behavioral and somatic issues that comprise major depressive disorder (MDD, or depression) are estimated to affect between 4.4 and 5.0% of people globally (2, 3). Biological and psychosocial factors can predispose individuals to developing MDD (4). Living with a chronic HIV infection, especially, significantly increases the risk of depression (5), with prevalence of depression amongst people living with HIV estimated to be as high as 50% (6, 7). Given the stigma associated with depression across cultures, people with depression may struggle to access mental healthcare resources, as Siyanda suggests. However, early referral and interventions have substantial benefits for the treatment and prevention of depression (8, 9). It is therefore imperative to continue to refine tools for early screening of depression and increase their accessibility in primary care clinics.

The Xhosa people comprise a diverse cultural group who speak variations of isiXhosa. The vast majority of Xhosa people live in the Eastern Cape and Western Cape provinces of South Africa (10). Over the last three centuries, European colonial expansion into the South African heartland occurred at the expense of indigenous populations, including Xhosa people, culminating in systematic disenfranchisement during the apartheid regime (11, 12). Under the apartheid government, especially, Xhosa people suffered severe marginalization as Afrikaans was the favored language in government and education in place of indigenous languages such as isiXhosa (13). As a result of these institutional barriers, it is difficult for people to access quality healthcare in isiXhosa today (14, 15). In order to reduce this healthcare inequity faced by Xhosa people, it is crucial that clinical resources are made available to them in their home language.

There is no word for “depression” in isiXhosa, but that does not mean that this debilitating illness is not found amongst the Xhosa people. In fact, the prevalence of depression in this group, estimated at 6.9%, is significantly higher than global estimates for depression in the general population (16). Substantial intra-group variability exists, with, for instance, a significantly higher prevalence rate of 31.9% amongst women in Khayelitsha during pregnancy and 12 weeks postpartum (17). Despite this, few isiXhosa-language tools exist for diagnosis of depression. An early study translated the Edinburgh Postnatal Depression Scale (EPDS) into isiXhosa and found satisfactory internal consistency (18), but this scale is limited in its target population. Another study validated an isiXhosa version of the Centre for Epidemiological Studies Depression Scale in a more general population group (16). The ongoing development and validation of the 16-item South African Depression Scale (SADS) in isiXhosa is particularly promising, especially for Xhosa people living with HIV (19, 20). However, the trade-off in developing such a tool is that limited comparisons can be made between scores from the SADS and other established scales until it is validated in other countries and cultures, thereby restricting synthesis of results across global studies.

The nine-item Patient Health Questionnaire (PHQ-9) is a useful measure for depression incidence and severity (21, 22). It has been validated as a diagnostic tool in clinical samples (23) as well as the general population (24), where it shows a high degree of convergent validity with other depression scales such as the Beck Depression Inventory (BDI). Furthermore, the PHQ-9 is easy to administer, especially in resource-limited settings (25), and exhibits similar results whether it is self-administered or carried out by an interviewer (26, 27). It is shown to be useful to screen for depression in both African populations (28) and people living with HIV (29, 30). Numerous validation studies for translations of the PHQ-9 have demonstrated its value as a depression scale in samples across the world (31–33). Given this global body of evidence, the PHQ-9 represents a measure of depression that could facilitate cross-cultural comparisons of depression pathology better than population- or geography-specific scales such as the EPDS or SADS. Despite these many advantages, to the best of our knowledge, an isiXhosa-language version of the PHQ-9 has not yet been validated and made publicly available. [Baron et al. (16) report findings from an isiXhosa version of the PHQ-9, but this version is not available alongside the study except by request to the authors.] Given the utility of the PHQ-9 in rapid screening of depressive symptomatology, a freely-available isiXhosa-language PHQ-9 may be an invaluable mental health triage tool for clinicians serving Xhosa people.

In this pilot study, we aim to produce and validate the first open-access isiXhosa-language version of the PHQ-9 depression scale. This version will be produced using a transcultural translation framework and administered to a cohort of adolescents living with HIV in Cape Town, South Africa. Responses on the PHQ-9 will be compared against those on the ASEBA Youth Self Report (YSR) forms as a gold standard. Our primary hypothesis is that the isiXhosa-language PHQ-9 will exhibit satisfactory reliability, measured as internal consistency using Cronbach's α. We also hypothesize that this translation of the PHQ-9 will show acceptable convergent validity (Pearson's correlation coefficient for PHQ-9 and YSR scores) and diagnostic accuracy (area under Receiver Operating Characteristic curve for PHQ-9 vs. YSR). This pilot study may pave the way for larger-scale validation studies of this isiXhosa-language PHQ-9 and add an easy-to-administer, culturally-specific questionnaire to the local clinician's toolbox.

## 2. Methods

### 2.1. Participants

Participants for this study will be recruited through the Adolescent Cognitive and Brain Imaging Study (“the GOLD study”) at Stellenbosch University and Tygerberg Hospital in Cape Town, South Africa. The GOLD study, which draws on the cohort of participants in the landmark CHildren with Early antiRetroviral therapy (CHER) trial, includes adolescents living with HIV who were initiated on antiretroviral therapy (ART) early in life (34). The CHER cohort is active and regularly willing to contribute to sub-studies, with as many as 80 children living with HIV and 80 age-matched HIV- controls participating in recent sub-studies (35).

#### 2.1.1. Inclusion Criteria

Inclusion criteria for participants will be: HIV+ status, currently receiving antiretroviral therapy, with plasma HIV RNA <40 copies per mL (indicating viral suppression), and speaking isiXhosa at home.

#### 2.1.2. Sample Size Estimation

*A priori* estimation of sample size in validation studies for psychometric tools is remarkably low (36). We utilized a web-based sample size calculation tool for reliability studies (37), available at this link, with the primary outcome of interest as the Cronbach's α reliability coefficient for the translated PHQ-9. The *a priori* sample size estimation showed that a sample size of *N* = 19 would be necessary to detect a Cronbach's α = 0.65 (indicative of a moderate reliability) at 80% power for the nine-item questionnaire. Therefore, we plan to recruit 20 participants for this pilot study.

#### 2.1.3. Ethical Considerations

We will receive written informed assent from participants and written informed consent from participants' parents or legal guardians in their home language for inclusion in the study before participation. The study will be conducted in accordance with the Helsinki Declaration and Good Clinical Practice (GCP) standards. The protocol for this study will be approved by the Stellenbosch University Human Research Ethics Committee (N21/10/116_Substudy N19/10/135).

### 2.2. Materials

#### 2.2.1. Patient Health Questionnaire (PHQ-9)

The English-language version of the PHQ-9 was designed with slight adaptations from the original version (21). The adaptations, intended primarily to improve comprehensibility of the scale to adolescents in 2022, were as follows: in item 7, we replaced “reading the newspaper” with “reading,” and in the final question, we replaced “if you checked off *any* problems” with “if you chose a number higher than 0.” The isiXhosa-language version of the PHQ-9 will be created from this English version using a transcultural translation framework (see Procedure). Both the English-language and isiXhosa-language versions of the PHQ-9 will be freely available in Supplementary Materials.

#### 2.2.2. Youth Self-Report (YSR) Form

The Achenbach System of Empirically Based Assessment (ASEBA) Youth Self-Report (YSR) form (38) measures behavioral issues representing syndromes such as “withdrawn/depressed,” “thought problems,” and “rule-breaking behavior.” The YSR has been validated as a measure of behavioral issues among adolescents (39), including in several studies in sub-Saharan Africa (40). In the current study, we will use data obtained from participants in the GOLD study during previous clinic visits when a validated bilingual (English and isiXhosa) YSR form was administered via an interview by a trained Research Assistant. During these visits, participants were asked (in the language in which they are most comfortable, English or isiXhosa) whether they think they have exhibited any of the behaviors in question over the past 6 months. These responses will be used as a gold-standard to compare with the translated version of the PHQ-9. Participants' most recent YSR scores will be used, and the YSR and PHQ-9 will be administered as closely as possible, and not more than 3 months apart. T scores for the “Withdrawn/Depressed” component within Syndrome Scales and “Affective Problems” component within DSM-Oriented Scales produced using YSR reponses will be used to determine participants' depressive symptoms and classify participants as “clinically depressed,” “borderline,” or “non-depressed” using ASEBA standards. No new YSR responses will be collected for this study; we will use data from the most recent clinic visit.

### 2.3. Procedure

#### 2.3.1. Setting

The study site for participant recruitment and data collection will be: Family Centre for Research with Ubuntu (FAMCRU), Ward J8, Tygerberg Hospital, Department of Paediatrics and Child Health, Faculty of Medicine and Health Sciences, Stellenbosch University. The basic infrastructure necessary for participant recruitment and data collection is already in place at FAMCRU.

#### 2.3.2. Transcultural isiXhosa Translation of the PHQ-9

The process of transcultural translation of psychometric tools involves steps to ensure that translated questionnaires remain accurate, relevant, and culturally acceptable (41, 42). We will adopt this systematic methodology to translate the PHQ-9 into isiXhosa using four steps:

1. Translation from English into isiXhosa by two independent bilingual (English and isiXhosa) speakers;
2. Review of isiXhosa translation by mental health experts and clinical care professionals who speak both languages;
3. Review of translation by a co-production panel involving adults living with HIV and adults with a lifetime history of depression; and
4. Blinded back-translation from isiXhosa into English by two additional independent bilingual translators.

The translated version will be refined after each step to preserve the core meaning and purpose of each item and incorporate culturally-specific idioms describing the affective components measured by the questionnaire where possible. Modifications will be reconciled in consultation with the translators and review panel involved in this process. The final isiXhosa-language version of the PHQ-9 will be available in Supplementary Materials.

#### 2.3.3. Co-production

Knowledge co-production, or participatory research methods, involves incorporating insights from individuals with lived experience of the conditions being studied. We will recruit 3-4 isiXhosa-speaking co-producers as a focus group to review and provide feedback on the isiXhosa translation of the PHQ-9 during the initial stages of this study. Up to two adults living with HIV and two adults who have experienced depression in their lifetime will be recruited through the FAMCRU clinic, Stellenbosch University network, and/or Twitter advertisements. These individuals will be invited to review the isiXhosa PHQ-9 after it has undergone review by a panel of experts and caregivers and suggest edits to improve the accessibility, comprehensibility, and cultural specificity of the translation. Co-producers will be reimbursed for their time.

#### 2.3.4. Participant Recruitment and Informed Consent

Participants will be recruited through the GOLD study at Tygerberg Hospital. Adolescents living with HIV who meet the current study's inclusion criteria will be contacted by FAMCRU research staff to ascertain interest in participation in this study. A trained counselor will discuss the study procedures in person with the potential participant's parent or legal guardian in their preferred language. Informed consent and assent forms will be available in two versions (English and isiXhosa). Parents or legal guardians of the participants will be asked to read and review the consent form. If participants agree to take part in the current study, written informed consent will be received from parents or legal guardians in advance of the study procedures. All participants will also be required to assent to the study procedures. Participants will be reimbursed R350 for their travel costs to visit the clinic for the study in line with Stellenbosch University policies.

#### 2.3.5. Psychometric Testing

Participants will be provided a private space for psychometric testing. A member of the research staff will brief participants on the procedures involved in the study and receive informed assent. The research staff will then confirm that the participants would prefer to respond to questionnaires in isiXhosa and offer them a choice between filling out the questionnaire themselves in writing or having the research staff ask them the questions verbally (for participants who may not be able to write or have a preference for spoken isiXhosa over written isiXhosa). If participants choose to complete the psychometric testing in writing, they will be asked to fill out a short demographic questionnaire and the translated version of the PHQ-9 in writing (paper and pencil). If participants choose to complete the questionnaire verbally, the member of the research staff will read each item on the questionnaire in a neutral manner and write down the response, without judgement or comment. In either case, the research staff member will be available to the participant for any clarifications to ensure participants understand what each question is asking. Once participants have completed the demographic questionnaire and the PHQ-9, the research team member will verify that all nine items of the PHQ-9 have been completed. If the questionnaire is incomplete, the participant's responses will be excluded. If the responses are complete, the research team member will calculate the total score on the PHQ-9 as the sum of the scores from individual items and input responses into a secure electronic data capture programme (Project RedCap), which includes built-in quality control checks. Data will be handled in accordance with the General Data Protection Regulation (GDPR) Act that informs best practice for personal data storage and transfer in Europe.

### 2.4. Statistical Approach

All statistical analyses will be performed using SPSS Statistics version 25 and R version 4.0.3. To determine the reliability of the isiXhosa PHQ-9, we will calculate Cronbach's α as a measure of internal consistency for the translated version. Inter-item and item-total score correlations will also be calculated. To determine convergent validity for the PHQ-9, Pearson's correlation coefficients will be calculated for the total PHQ-9 scores and T scores for the “Anxious/Depressed” and “Withdrawn/Depressed” syndrome scales on the YSR. To determine the criterion validity of this PHQ-9 version, we will compare participant responses on the isiXhosa PHQ-9 with the YSR forms and calculate diagnostic sensitivity and specificity, predictive values (PPV/NPV), likelihood ratios (PLR/NLR), and Youden's Index. A Receiver Operating Characteristic (ROC) curve will be produced for the isiXhosa PHQ-9 and area under the curve (AUC) will be calculated for the PHQ-9 to determine diagnostic performance in comparison to the YSR. Finally, individual item analyses will be conducted to determine whether mean scores on each item of the PHQ-9 differed between depressed, borderline, and non-depressed participants (as determined by the YSR responses).

## Data Availability Statement

Data supporting the conclusions of this article will be made available by the authors, without undue reservation.

## Ethics Statement

The studies involving human participants were reviewed and approved by Stellenbosch University Human Research Ethics Committee (HREC), Study Reference N21/10/116_Sub Study N19/10/135. Written informed consent to participate in this study was provided by the participants' legal guardian/next of kin.

## Author Contributions

AMRL and BL conceptualized the study together. AMRL designed the study and statistical approach, and wrote the initial draft. BL refined experimental design and reviewed the manuscript. Both authors contributed to the article and approved the submitted version.

## Funding

This study is supported by funding from the Wellcome Trust (Grant No. 218493/Z/19/Z, Translational Neuroscience Ph.D. Programme) and the Harold Hyam Wingate Foundation (Medical Research Travel Grant) awarded to AMRL. Infrastructure for this study was made possible by NIH Grant R01HD099846 supporting the GOLD study.

## Conflict of Interest

The authors declare that the research was conducted in the absence of any commercial or financial relationships that could be construed as a potential conflict of interest.

## Publisher's Note

All claims expressed in this article are solely those of the authors and do not necessarily represent those of their affiliated organizations, or those of the publisher, the editors and the reviewers. Any product that may be evaluated in this article, or claim that may be made by its manufacturer, is not guaranteed or endorsed by the publisher.
